# Large Outbreak Caused by Methicillin Resistant *Staphylococcus pseudintermedius* ST71 in a Finnish Veterinary Teaching Hospital – From Outbreak Control to Outbreak Prevention

**DOI:** 10.1371/journal.pone.0110084

**Published:** 2014-10-15

**Authors:** Thomas Grönthal, Arshnee Moodley, Suvi Nykäsenoja, Jouni Junnila, Luca Guardabassi, Katariina Thomson, Merja Rantala

**Affiliations:** 1 Central Laboratory, Department of Equine and Small Animal Medicine, University of Helsinki, Helsinki, Finland; 2 Department of Veterinary Disease Biology, Faculty of Health and Medical Sciences, University of Copenhagen, Copenhagen, Denmark; 3 Food and Feed Microbiology Research Unit, Finnish Food Safety Authority Evira, Helsinki, Finland; 4 4Pharma Ltd., Espoo, Finland; 5 Veterinary Teaching Hospital, University of Helsinki, Helsinki, Finland; Columbia University, United States of America

## Abstract

**Introduction:**

The purpose of this study was to describe a nosocomial outbreak caused by methicillin resistant *Staphylococcus pseudintermedius* (MRSP) ST71 SCC*mec* II-III in dogs and cats at the Veterinary Teaching Hospital of the University of Helsinki in November 2010 – January 2012, and to determine the risk factors for acquiring MRSP. In addition, measures to control the outbreak and current policy for MRSP prevention are presented.

**Methods:**

Data of patients were collected from the hospital patient record software. MRSP surveillance data were acquired from the laboratory information system. Risk factors for MRSP acquisition were analyzed from 55 cases and 213 controls using multivariable logistic regression in a case-control study design. Forty-seven MRSP isolates were analyzed by pulsed field gel electrophoresis and three were further analyzed with multi-locus sequence and SCC*mec* typing.

**Results:**

Sixty-three MRSP cases were identified, including 27 infections. MRSPs from the cases shared a specific multi-drug resistant antibiogram and PFGE-pattern indicated clonal spread. Four risk factors were identified; skin lesion (OR = 6.2; CI_95%_ 2.3–17.0, *P* = 0.0003), antimicrobial treatment (OR = 3.8, CI_95%_ 1.0–13.9, *P* = 0.0442), cumulative number of days in the intensive care unit (OR = 1.3, CI_95%_ 1.1–1.6, *P* = 0.0007) or in the surgery ward (OR = 1.1, CI_95%_ 1.0–1.3, *P* = 0.0401). Tracing and screening of contact patients, enhanced hand hygiene, cohorting and barrier nursing, as well as cleaning and disinfection were used to control the outbreak. To avoid future outbreaks and spread of MRSP a search-and-isolate policy was implemented. Currently nearly all new MRSP findings are detected in screening targeted to risk patients on admission.

**Conclusion:**

Multidrug resistant MRSP is capable of causing a large outbreak difficult to control. Skin lesions, antimicrobial treatment and prolonged hospital stay increase the probability of acquiring MRSP. Rigorous control measures were needed to control the outbreak. We recommend the implementation of a search-and-isolate policy to reduce the burden of MRSP.

## Introduction

Methicillin-resistant *Staphylococcus pseudintermedius* (MRSP) has emerged as a major animal pathogen in veterinary medicine [1], similar to methicillin resistant *Staphylococcus aureus* (MRSA) in human medicine [2]. MRSP can cause a wide variety of infections that are difficult to treat due to multi-drug resistance [1]. Transmission is mainly due to global spread of epidemic clones such as ST71, the predominant clone in Europe, and ST68, the predominant clone in North America [3]. Among others, hospitalization and antimicrobial treatment have been recognized as risk factors for MRSP colonization or infection [4]–[7]. Indistinguishable or closely related MRSP isolates from patients, environmental sites and staff members at veterinary clinics have been reported [8], [9] suggestive of veterinary care associated spread of MRSP. However, to our best knowledge, no nosocomial outbreak reports of MRSP have yet been published. In addition, little information exists about infection control and preventive measures in practical situations.

**Figure 1:**
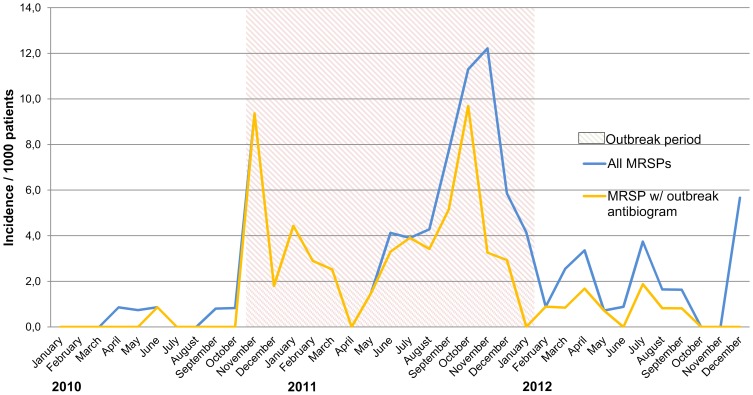
The monthly cumulative incidence of all MRSPs and MRSPs displaying the outbreak antibiogram (MRSP ST71) among patients of the Small Animal Hospital of Helsinki University from January 2010 to December 2012. In late 2011 a small cluster of ST45 among hospitalized patients contributed to an increase in incidence. From January 2012 onwards the great majority of new MRSP findings have been detected in screening targeted to risk patients on admission. In December 2012 the increase was not due to a cluster, but was due to the detection of different types of MRSPs mainly in patients belonging to risk groups.

The Veterinary Teaching Hospital of the University of Helsinki experienced a nosocomial MRSP outbreak between November 2010 and January 2012 with 63 confirmed cases among canine and feline patients. Prior to this MRSP was a very rare finding among patients of the Veterinary Teaching Hospital (Figure 1). The goal of this study was to (i) identify and characterize the strain causing the outbreak, (ii) to describe the outbreak and determine risk factors for acquiring MRSP, (iii) to describe the control measures implemented to contain the outbreak and (iv) to present our current policy for prevention of further outbreaks and the spread of MRSP. The hypothesis was that in addition to previously recognized risk factors, many variables related to patient condition, duration of surgical procedures, as well as the length of antimicrobial therapy increases the risk for MRSP acquisition during an outbreak.

## Materials and methods

### The hospital setting

The Veterinary Teaching Hospital of the University of Helsinki is a national primary care and referral animal hospital in Finland. The hospital provides 24/7 emergency and intensive care services for animals primarily in the Greater Helsinki area. The Small Animal Hospital of the unit has approximately 18 000 visits annually, with nearly 2000 surgical procedures. Approximately 80% of patients are dogs, 17% cats and the rest are other species. Bacteriological specimens from the hospital are investigated by the Clinical Microbiology Laboratory of the Faculty of Veterinary Medicine. The laboratory receives specimens from all over Finland. Apart from investigation of clinical specimens the laboratory is responsible for resistance surveillance of small animal pathogens in the hospital and in Finland.

### Epidemiological investigation and definitions

The study population consisted of dogs and cats that had been hospitalized for 1 day or more at the Small Animal Hospital during the outbreak period (November 2010 – January 2012) and thus were potentially exposed to nosocomial MRSP. Cases were either colonized (MRSP cultured only from mucous membranes) or infected (MRSP cultured from an infection site) with MRSP displaying the following antibiogram; resistance to oxacillin (and thus all beta-lactams), erythromycin, clindamycin, sulfamethoxazole-trimethoprim, gentamicin, tetracycline and enrofloxacin and susceptibility to fusidic acid and amikacin. To exclude community acquired MRSPs, only infections detected in the outbreak period either after surgical procedures performed at the hospital or other infections which appeared after prolonged or several treatment periods in the hospital were included. Colonized patients were enrolled if the MRSP was detected after at least 1 day of hospitalization and the animal had been treated in the same wards as MRSP positive patients. Controls were patients from the same population as cases but were negative in MRSP screening. Patients with a positive MRSP specimen on first admission, and non-hospitalized (polyclinic) patients were excluded from the study.

The investigation extended over a total of 26 months comprising the outbreak period (November 2010 – January 2012) and the follow-up period (February 2012 – November 2012). Treatment histories of the patients were gathered from the hospital patient registry software (Provet YES 1.1, Finnish Net Solutions Oy, Finland). Variables, including their definitions, are presented in Table 1. Data were collected for each individual from one month prior to the index case (i.e. from October 2010) up until the first positive MRSP finding (cases) or the latest date the patient was screened negative for MRSP (controls). Dates refer to when the specimen was taken. In addition, data on the cumulative incidence of MRSP before, during and after the outbreak was collected and presented as total incidence (all new MRSP findings) and as incidence of the outbreak MRSP. This data were extracted from the laboratory information system (connected to the patient registry software). Since one patient with the defined MRSP had been observed in July 2010, a trace-back analysis was performed to evaluate any relationship of that patient to the current outbreak.

**Table 1:** Variables analyzed from cases and controls during the MRSP outbreak in the Small Animal Hospital of Helsinki University between 2010 and 2011.

Species (dog/cat)	Emergency surgery (during weekend/evening/night)	Aminopenicillin medication given
Age (years)	Length of anesthesia (min)	bDays of aminopenicillin therapy
Gender	bDays in hospital	Cephalosporin medication given
Breed	bDays in surgery ward	bDays of cephalosporin therapy
Weight (kg)	bDays in intensive care unit	cEnrofloxacin medication given
aSeverity of condition	bDays in other wards	bDays of enrofloxacin therapy
Skin lesions of any cause	Antimicrobial medication given	Proton pump inhibitor (PPI) given
Surgical procedure	bDays of any antimicrobial therapy	bDays of PPI therapy

a Severity was judged by the author (TG) on a scale of 1 to 5 after reviewing the patient record on admission and was based on the guidelines provided by the American Society of Anesthesiologists.

b The same patient might have had several visits or courses of medication, therefore the cumulative number of days for these variables was recorded until the first positive MRSP specimen (cases), or latest negative MRSP specimen (controls), see text for details.

c Enrofloxacin was the only fluoroquinolone used for these patients.

This study did not require separate ethical approval since outbreaks are routinely investigated according to the hospital ethical guidelines to ensure patient safety. The owners of the animals were informed about the outbreak and study. They agreed to the investigation as well as any attempts to control the outbreak. Owners also gave permission to take the necessary specimens. The hospital covered the costs of screening specimens of exposed patients and specimens to monitor the efficacy of the control measures. Data was handled anonymously.

### Data analysis and statistical methods

Descriptive analysis of cases was done by presenting the number of new cases per week over the outbreak and follow-up periods in the epidemic curve along with the implemented control measures. The number of colonized and infected patients was recorded. The attack rate was determined by using the number of hospitalized patients as the denominator. The risk factors (Table 1) for acquiring MRSP were assessed with logistic regression. For the risk factor study data were available for 55 cases and 213 controls. Each factor was first modeled using a univariable logistic regression models. To control for confounders, a stepwise multivariable logistic regression analysis was conducted for the risk factors with a *P* value ≤0.05 in the univariate analyses. In the stepwise selection process, a significance level of 0.15 was required to allow a variable into the multivariable model, and a significance level of 0.20 was required for a variable to stay in the multivariable model. Odds ratios (OR) with 95% confidence intervals (CI) were calculated. *P* values (Wald) ≤0.05 were considered statistically significant. All statistical analyses were done using SAS System for Windows, version 9.3 (SAS Institute Inc., USA).

### Microbiological investigation

**Figure 2:**
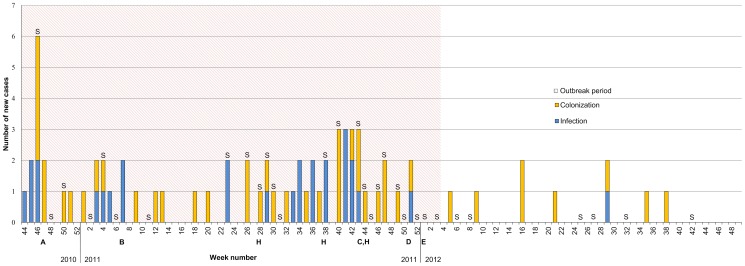
An epidemic curve showing new MRSP ST71 cases during the outbreak in 2010–2012 at the Small Animal Hospital of Helsinki University. The outbreak period was between November 2010 and January 2012, after which the follow-up period was started. A: hospital closed for 2 days for cleaning and disinfection, B: establishment of cohort ward, C: nurse responsible for hospital hygiene appointed, D: hospital closed for 5 days for cleaning and disinfection, E: veterinarian appointed as infection control officer. S: Screening of hospitalized patients, H: environmental swabs taken.

Specimens for bacterial cultures were taken from infection sites of all patients as soon as signs of infection were noticed. To screen for MRSP colonization (screening specimens), specimens were taken from the mucous membranes of patients with or without infection. For this, three sites were swabbed in patient; the nares and oral mucous membranes with one swab, and the perineum with another. If the patient had a wound or skin sore, that was also swabbed. Screening specimens were taken frequently from contact patients and regularly from all hospitalized patients (Figure 2) in order to monitor the extension of the outbreak and efficacy of control measures. Patients were screened repeatedly if they had long term hospitalization or several treatment periods. In the autumn 2011 there was a two month period of enhanced surveillance when every hospitalized patient (n = 72) was screened both on admission and on discharge. In addition, environmental swabs (n = 65) were taken to evaluate efficacy of daily cleaning and disinfection routines and the role of the environment as the source of MRSP on three occasions.

Specimens from superficial infection sites and urine were cultured aerobically, whereas specimens from deep lesions, aspirates and blood were also cultured anaerobically. Both non-selective and selective plates were used for primary cultures according to the laboratory protocol. The protocol also included direct plating onto MRSA selective agar (MRSA Select, Bio Rad Laboratories, France) and enrichment culture for MRSP (see below). Screening specimens from the patient were cultured by pooling the swabs into an enrichment broth (Brain Heart Infusion broth with 6.5% NaCl, Tammer-Tutkan Maljat Oy, Finland) and incubated for 16–22 h at +35.0°C (±0.2°C). The enrichment broth was then plated onto MRSA-selective agar and incubated up to 48 h at +35.0°C (±0.2°C), and were interpreted once a day. The limit of detection for enrichment culture method had been determined to be ≥10 CFU for MRSP with oxacillin minimum inhibitory concentration (MIC) of ≥4 µg/ml in internal validation. Suspected MRSP colonies (pale pink to pink colonies) were subcultured onto tryptic soy agar with 5% sheep blood (Oxoid Ltd., UK). Presumptive identification of *S. pseudintermedius* was based on typical colony morphology, positive tube coagulation test (BBL Coagulase Plasma, Becton Dickinson, USA) and susceptibility to polymyxin B (300 U, Oxoid Ltd, UK) (sensitive ≥10 mm, resistant <10 mm). If identification was doubtful, sugar fermentation tests (Diatabs, Rosco Diagnostica A/S, Denmark) or API Staph ID 32 (bioMérieux SA, France) were used. Antimicrobial susceptibility testing was done in accordance with Clinical and Laboratory Standards Institute guidelines [10] by using the disk-diffusion method (Oxoid Ltd., UK). Breakpoints for oxacillin susceptibility presented by Bemis et al. [11] were used. Oxacillin MIC was determined by E-test (Oxoid Ltd., UK). MRSP isolates were sent to the Finnish Food Safety Authority (Evira) for verification of the presence of *mec*A [12].

### Isolate characterization

**Figure 3:**
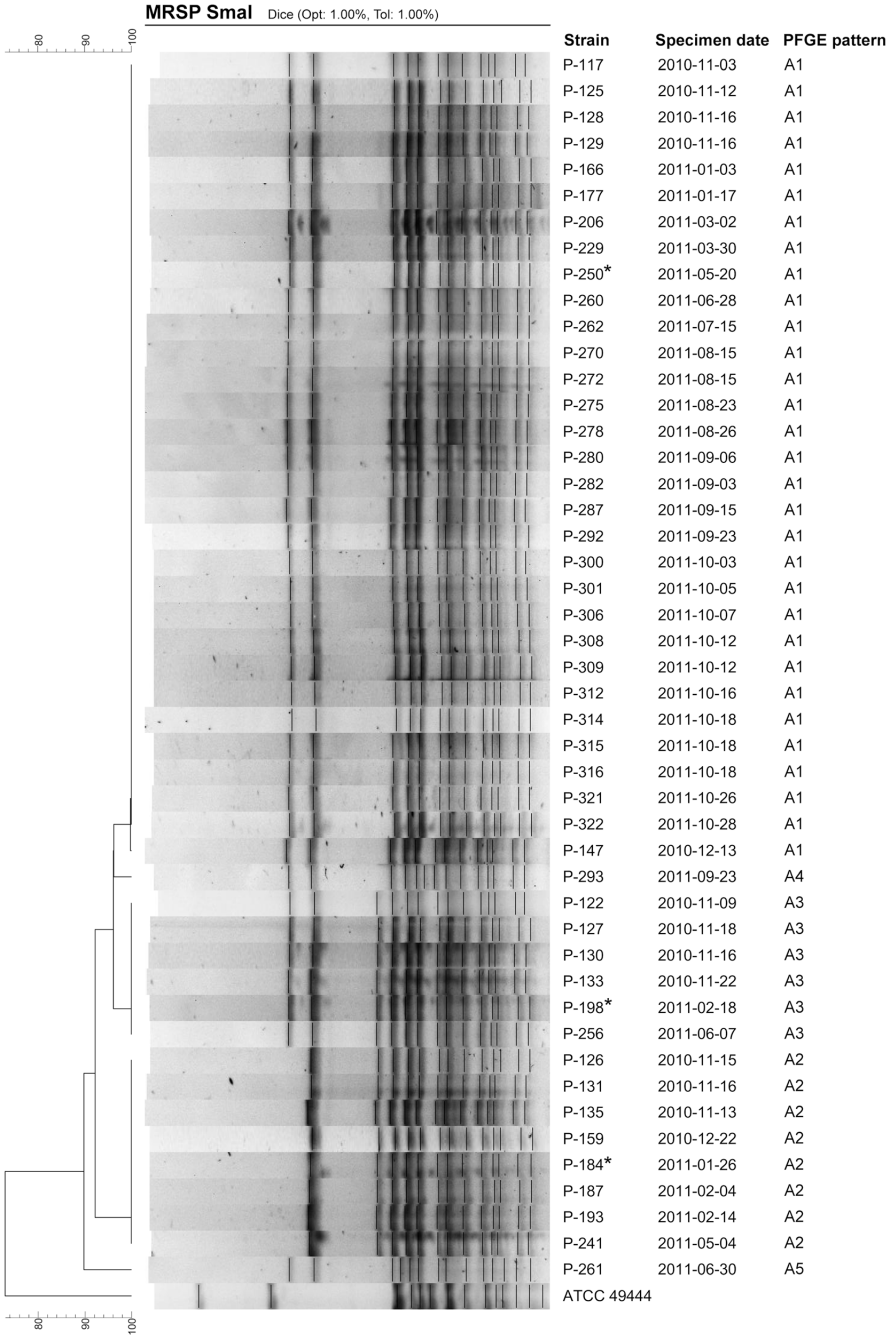
Dendrogram of 47 MRSP isolates with the outbreak antibiogram (see text). *Staphylococcus pseudintermedius* ATCC 49444 is displayed as a control. *Further characterized by multilocus sequence typing and SCC*mec*-typing.

Forty-seven isolates were available for pulsed field gel electrophoresis (PFGE) typing. A modified version of the HARMONY protocol as described by Murchan et al. [13] was used. Approximately 4×10^8^ colony forming units per strain were suspended into 200 µl of EC-buffer (1 M sodium chloride, 0,5% Polyoxyethylene 20 cetyl ether, 0,2% w/v sodium deoxycholate, 0,5% w/v N-lauroyl-sarcosine sodium salt, 0,1 M EDTA, 6 mM 1,0 M Tris-HCl). The plugs were made by mixing the bacteria and EC-buffer suspension with 20 µl of lysostaphin (1 mg/ml, Sigma-Aldrich, USA), and 200 µl of 2% SeaPlaque GTG agarose (Lonza Inc., USA). The digestion was done using a 10% NEBuffer 4 and 20 U SmaI (New England BioLabs Inc., USA) for 4–18 hours. The pulse field electrophoresis was done in 1% SeaKem agar (Lonza Inc., USA) on the CHEF-DR III system (Bio-Rad Laboratories, USA). Gels were stained with SYBR Safe DNA gel stain (Life technologies, USA) and analyzed using GelCompar II v. 6.5 software (Applied Maths NV, Belgium), and cluster analysis was performed by UPGMA based on the Dice similarity coefficient, with optimization and position tolerance both set at 1%. Isolates were clustered using an 85% similarity cut-off. The strains were considered to be closely related (≤3 band differences) or subtypes of the same clone (4–6 band differences) according to Tenover *et al*. [14]. Based on PFGE, three strains representing subtypes of the clone (Figure 3) were further characterized by SCC*mec* [15] and multilocus sequence typing (MLST) [16], and confirmed to be *S. pseudintermedius* by species specific *nuc* PCR [17]. MLST type was determined by comparing sequences of the housekeeping genes to the *S. pseudintermedius* MLST database (http://pubmlst.org/spseudintermedius/).

## Results

### Description of the outbreak

During the outbreak period 63 cases were identified; 27 (43%) of these were infections, while 36 (57%) were colonized patients. Of the infected patients, three developed MRSP infection several weeks after colonization was detected. The types of the MRSP-infections are summarized in Table 2. The attack rate of MRSP among hospitalized patients was 2.1% (63/2969) and among patients discharged from the ICU 3.8% (43/1121). MRSP was the cause of a surgical wound infection in 0.9% of surgical procedures (17/1864). Fifty-eight of the cases (92%) were dogs and five (8%) were cats; dogs represented more than 40 different breeds, all five cats were domestic short haired. The epidemic curve indicating the number of new cases per week is presented in Figure 2; and the cumulative incidences both for the outbreak MRSP and all new MRSP findings before, during and after the outbreak in Figure 1.

**Table 2:** Nosocomial infections (n = 27) caused by the MRSP outbreak strain (ST71, SCC*mec* II–III) in the Small Animal Hospital of Helsinki University between 2010 and 2011.

Infection type	Number of infections
Surgical site infections (total)	19
Required surgical revision	3
Involved orthopedic devices^1^	7
Others (uncomplicated)	9
Other wound infections	3
Otitis^2^	1
Bite wound^3^	2
Dermatitis^4^	1
Cystitis complicated by uroliths^5^	1

1 Some cases required removal of surgical devices and revision.

2 Patient had orthopedic surgery and several visits to the hospital, otitis was subsequently diagnosed.

3 Both patients presented with severe bite wounds; after prolonged hospital stay MRSP was cultured from the wound.

4 Patient presented with pneumonia, autoimmune myositis and dermal vasculitis; later developed MRSP infection on the skin lesion.

5 Colonization with MRSP preceded the cystitis.

The index patient was a 3 year-old dachshund that was referred to the Small Animal Hospital emergency care unit at the end of October 2010. The dog had systemic inflammatory response syndrome with disseminated intravascular coagulation due to necrotizing mastitis and a postoperative complication after cesarean section and ovariohysterectomy performed at two different private practices. The dog required surgery again at the Small Animal Hospital to remove necrotized tissue and was treated in the intensive care unit (ICU) for 1 week. The tissue specimen yielded pure growth of *Escherichia coli*. However, two days after discharge, in the beginning of November 2010, a surgical site infection was noted. The bacteriological culture revealed MRSP with the aforementioned multi-drug resistance antibiogram. The finding was extraordinary and therefore an outbreak investigation was initiated. This involved active case finding by culturing all infection sites and screening of patients potentially exposed to MRSP.

Trace-back analysis to the case in June 2010 did not reveal any apparent relationship to the outbreak. Subsequently the index case, active case finding revealed many new cases (Figure 2). After control measures in late 2010, and early 2011 the incidence of MRSP decreased for a while. The situation worsened again in the summer and fall of 2011, leading to extensive control measures after which control was achieved. However, the investigation was interfered by a small cluster of MRSP ST45 detected in late 2011(Figure 1, File S2). The outbreak was considered to be over in January of 2012, when no new MRSP findings were revealed in three consecutive MRSP screenings of all hospitalized patients. After this the follow-up period was started. During the follow up period nine new MRSP findings with the outbreak antibiogram were detected. Of these, seven were explained by previous exposure due to hospitalization during the outbreak. Thus the total toll of cases connected to the outbreak was 70. The other two were not spatially or temporally connected to the outbreak. In the follow-up period all MRSP cases with the outbreak antibiogram were identified on admission using the risk patient classification criteria (Table 3). Regardless of this, all hospitalized patients were screened for MRSP on seven occasions, but no new cases were detected among these (Figure 2).

**Table 3:** The current risk based classification of patients at the Small Animal Hospital of Helsinki University and resulting measures.

Classification	Criteria (any of the following)	Example of measures
High risk patients	MRSP-positive	Treated in cohort ward
	Has been hospitalized >24 hours and has signs of a hospital acquired	Barrier nursing
	infection	Surgery at the end of the day
		Disinfection of facilities
		Infection sites cultured
		Standard precautions*
Medium risk patients	Has a history of recurrent ear or skin infection	Screened for MRSP
	Has a history of prolonged or numerous hospital visits or visits at	Treated in separate rooms
	other veterinary clinics	reserved for medium risk
	Has a history of prolonged or numerous antimicrobial treatments	patients
	Has been exposed to a patient with MRSP	Surgery at the end of the day
	Has had surgery elsewhere and has a surgical site infection	Standard precautions
	Has a suppurative wound infection	Infection sites cultured
Low risk patients	All other patients	All other rooms
		Standard precautions

*Includes hand disinfection, hygienic work routine, and use of protective clothing in case of dirty procedures.

### Risk factor analysis

Several risk factors were significant by univariable analyses (Table 4, Table S1). However, after controlling for confounders, the logistic regression model revealed only four significant risk factors; skin lesions of any origin (including surgical incisions) (OR 6.24, CI_95%_ 2.30–16.97), antimicrobial therapy regardless of duration (OR 3.80, CI_95%_ 1.04–13.92), cumulative number of days spent in the ICU (OR 1.33, CI_95%_ 1.13–1.57) or in the surgery ward (OR 1.13, CI_95%_ 1.01–1.27). The results of the univariable and multivariable analyses are presented in detail in Table 4.

**Table 4:** Risk factors associated with acquisition of MRSP during the outbreak in the Small Animal Hospital of Helsinki University between 2010 and 2011.

					Univariable logistic regression		Multivariable logistic regression	
Binary variables	MRSP-pos		MRSP-neg		Unadjusted OR	Wald	Adjusted OR	Wald
	(*n* = 55)		(*n* = 213)		(95% CI)	*P*	(95% CI)	*P*
	n	%	n	%				
**Demographics**								
Gender: M vs. F	30	54.5	96	45.1	1.46 (0.80–2.66)	0.212		
Species: dog vs. cat	50	90.9	192	90.1	1.09 (0.39–3.06)	0.864		
**Epidemiological data**								
Skin lesion	49	89.1	85	39.9	12.40 (5.06–30.37)	**<0.001**	6.24 (2.30–16.97)	**0.0003**
Antimicrobial treatment	52	94.6	130	61.0	11.07 (3.33–36.79)	**<0.001**	3.80 (1.04–13.92)	**0.0442**
Surgical procedure	45	81.8	67	31.5	9.81 (4.65–20.70)	**<0.001**		
Cephalosporin treatment	21	38.2	24	11.3	5.10 (2.54–10.26)	**<0.001**		
Enrofloxacin treatment	18	32.7	33	15.5	2.70 (1,3–5.2)	**0.005**		
Severity (1 vs. others)	46	85.5	8	14.5	2.84 (1.3–6.4)	**0.012**		
Aminopenicillin treatment	34	61.8	93	43.7	2.09 (1.14–3.85)	**0.018**		
Treatment in ICU	41	74.6	126	59.2	2.02 (1.03–3.95)	**0.039**		
Proton pump inhibitor treatment	36	65.5	114	53.5	1.65 (0.89–3.06)	0.115		
Orthopedic vs. soft tissue surgery	20	44.4	20	30.3	1.84 (0.83–4.08)	0.132		
Other antimicrobial treatment	12	21.8	32	15.0	1.58 (0.75–3.33)	0.229		
Emergency surgery	6	13.6	9	13.4	1.02 (0.33–3.13)	0.976		

Continuous variables	MRSP-pos		MRSP-neg		Unadjusted OR	Univariate	Adjusted OR	Multiple regression
	(*n* = 55)		(*n* = 213)		(95% CI)	*P*	(95% CI)	*P*
	n	mean	n	mean				
**Demographics**								
Bodyweight	55	20.7	201	19.2	1.01 (0.99–1.03)	0.493		
Age	55	4.8	212	4.9	1.01 (0.92–1.10)	0.791		
**Epidemiological data**								
Cum. days in ICU	55	2.9	213	1.5	1.39 (1.20–1.61)	**<0.001**	1.3(1.1–1.6)	**0.0007**
Cum. days in surgery ward	55	3.4	213	1.1	1.28 (1.15–1.42)	**<0.001**	1.1 (1.0–1.3)	**0.0401**
Cum. days in hospital (all wards)	55	7.2	213	4.5	1.15 (1.08–1.24)	**<0.001**		
Cum. days of proton pump inhibitors	36	5.5	114	2.9	1.08 (1.01–1.17)	**0.037**		
Length of surgical procedure (10 min change)	41	203.2	57	170.9	1.04 (0.99–1.10)	0.080		
Cum. days in other wards	55	2.0	213	2.6	0.92 (0.81–1.05)	0.210		
Cum. days antimic. given	55	12.0	213	5.4	1.01 (0.99–1.02)	0.364		
Cum. days of enroflox.	18	6.5	33	4.7	1.03 (0.96–1.11)	0.459		
Cum. days of aminopen.	34	13.9	93	10.1	1.00 (0.99–1.02)	0.481		
Cum. days cephalosp.	21	5.3	23	4.1	1.03 (0.93–1.15)	0.518		
Cum. days of other antimic.	12	4.7	32	6.7	0.98 (0.91–1.06)	0.614		

OR =  Odds Ratio, CI =  Confidence Interval.

### Characterization of the outbreak strain

PFGE analysis supported that the outbreak was due to clonal spread of MRSP. Isolates clustered to one dominant pulsotype, A1 (n = 31), and four subtypes; A2 (n = 8), A3 (n = 6), A4 (n = 1) with a one band difference and A5 (n = 1) with a four band difference (Figure 3). On the basis of the typing results of the three isolates, the strain responsible for the outbreak belonged to ST71 (File S1) and harbored SCC*mec* II–III.

### Outbreak control measures

The staff and students were informed by e-mail about the situation on numerous occasions and training sessions were organized. The use of alcohol-based hand rubs before and after every patient contact was emphasised, and the use of protective gear (gloves and gowns) was required during dirty procedures (i.e. treatment of wounds, performing ear flushing, dental procedures or administering enemas), or when handling MRSP patients. The compliance to follow hygienic work order (i.e. performing clean procedures prior to dirty ones and examining healthy patients before diseased) was enhanced and immediate disinfection of secretions with 1% Virkon S (Antec International, UK) was demanded. The efficacy of the control measures were surveyed by frequent screening of hospitalized patients (Figure 2). Sixty-five environmental swabs from high-touch surfaces were collected. MRSP with the outbreak antibiogram was detected in only one environmental specimen, and originated from the cohort ward where MRSP patients were treated since February 2011. This ward was established to house MRSP-positive and high-risk patients (Table 3). Extensive cleaning and disinfection of all hospital surfaces were undertaken a few weeks after the first case (Figure 2). The ICU was closed during this time. Surface disinfection with a 1% Virkon S solution was increased.

In November 2011, a nurse responsible for hospital hygiene was appointed allowing a more effective tracking of discharged patients exposed to MRSP. These patients received a “MRSP exposed” tag in the electronic patient record. The tag was a sign for staff to screen the patient for MRSP and classify it as a medium-risk patient upon returning to the hospital (Table 3). Prior to the end of 2011 the hospital, excluding the emergency policlinic, was closed for five days for large scale cleaning and disinfection. All staff participated in the cleaning. From the beginning of 2012 a veterinarian was appointed as infection control officer to enforce prudent use of antimicrobials and consult in hospital hygiene and patients involving infections. After these measures control of the outbreak was finally achieved (Figure 2).

A “search-and-isolate” policy was launched in early 2012 to prevent further outbreaks and the spread of MRSP within the hospital and to the community. In addition to standard precautions (hand disinfection, hygienic work routine, and use of protective clothing in case of dirty procedures) this includes (1) the risk based classification of all patients (Table 3), (2) screening of patients at risk (at the expense of the owner), (3) isolation of high risk and MRSP positive patients, (4) screening of contact patients of new cases either in the hospital or upon revisit (at the expense of the hospital), (5) early initiation of the outbreak investigation, (6) surveillance and bacteriological sampling of treatment associated infections and (7) prudent use of antimicrobials.

## Discussion

The MRSP outbreak spanned over a period of 14 months, during which 63 patients were found to be infected (n = 27) or colonized (n = 36). Additionally, seven more temporally and spatially connected cases were detected during the follow up period. There are several factors which suggest that this was a nosocomial outbreak: (i) the cases were spatially and temporally connected, (ii) the patients had no evidence of MRSP on admission and (iii) molecular characterization supported clonal spread. Also, all infections were related to hospital care as they were surgical site infections or other infections which appeared after prolonged hospital treatment. It was likewise considered very unlikely, that MRSPs of colonized patients were community acquired since this MRSP type was very rare prior to the outbreak and no similar type of MRSP was observed among outpatients or specimens submitted from private clinics during the outbreak. In addition, many of our cases (n = 30) had given a negative MRSP result in former bacteriological specimens taken on or soon after the first admission.

This is the first report of MRSP in Finland. The outbreak strain was the multi-drug resistant global MRSP clone ST71-SCC*mec* II–III [3]. This clone has also been found in other Nordic countries such as Denmark [3], Sweden [18] and Norway [19]. For our patients the strain caused a number of nosocomial infections ranging from dermatitis to osteomyelitis, with the majority being surgical site infections after non-elective procedures. In one case the colonization was followed by a urinary tract infection, complicated by urolith formation leading to surgery. In another case an MRSP infection was the most likely cause for euthanasia, but this could not be confirmed since no autopsy was done.

Many patients required prolonged hospital treatment or surgical procedure to combat the infection. Majority of infections were treated without systemic antimicrobials, but if considered necessary, amikacin was used. The exception was a case with urinary tract infection which was treated with nitrofurantoin. The fact that MRSP infections were manageable without systemic antimicrobials is encouraging. This approach could even be considered in infections caused by susceptible bacteria, provided that no systemic signs are present.

The risk factors for MRSP according to the multivariable analysis were skin lesions of any origin, antimicrobial therapy – regardless of duration, and cumulative number of days treated in the ICU or in the surgery unit. The length of antimicrobial therapy was not a significant risk factor in the final model. This indicates that any antimicrobial treatment may increase the risk for the acquisition of MRSP in an outbreak, possibly due to the high infection pressure. The result may not be generalized to the outpatient population, in which the cumulative use of antimicrobials could be the more important factor. Multiple factors operating in an intricate fashion lead to the elimination of many of the studied variables in the final model, suggesting the presence of confounders, such as surgery and skin lesions, and surgery and the use of antimicrobials.

In hospital outbreaks caused by multidrug resistant bacteria, it is often expected to have more colonized than infected patients, as were also the case in this outbreak. While infection is more harmful to the individual patient and more expensive to the hospital, failure to recognize colonized patients would likely have led to an underestimation of the extent of the outbreak, or even the unrecognition of the outbreak. Colonized and infected patients were pooled as cases for the risk factor analysis. If handled separately in the risk factor analysis one would not possibly reach the power to identify a common source or relevant factors associated with emergence of the pathogen. We think it is reasonable to assume that there is no biological difference in the acquisition of MRSP in colonized and infected patients. Colonization can precede the infection, as was the case also in our study in three of the patients, or colonization and infection may develop simultaneously depending on where the pathogen enters the body as well as on the characteristics of the individual's immune status. In many cases infection never occurs. Also, exact differentiation between colonization and infection especially in mild cases can be difficult, since signs of inflammation can be very similar to infection.

Studies evaluating risk factors for MRSP have previously been done by comparing patients diagnosed with MRSP infection with patients with methicillin susceptible *S. pseudintermedius* (MSSP) infection [6], [7], or comparing MRSP positive patients with MRSP negatives on admission to a hospital [5]. Antimicrobial treatment [5], [6] as well as treatment duration [4] has been associated with an increased risk for MRSP. Interestingly, Lehner *et al.* [7] did not observe antimicrobial treatment as a risk factor for MRSP infection, but linked glucocorticoid therapy to an increased risk for MRSP infection. However, glucocorticoid therapy may also be associated with other factors, such as atopy or allergy [5]. Risk factors regarding MRSP in cats are poorly documented, but there is evidence that colonization rates of *S. pseudintermedius* [20] and MRSP are lower in cats compared to dogs [21]. Conversely, Lehner *et al.* [7] concluded that cats were at an increased risk of MRSP infection compared to dogs, although the result may have been due to bias caused by the sampling strategy. In our study the species was not a risk factor for MRSP. Cats were not separately analyzed in our study due to their low number. Still, species specific risk factors warrant further study.

Previous hospitalization has been shown to be a risk factor for MRSP [5], [7], suggesting that MRSP is an important hospital associated pathogen. The epidemiology of MRSP appears to be comparable to that of MRSA in humans or animals, as also MRSA originally emerged in hospitals [1], [22]. Other groups at risk for MRSP are patients with chronic dermatological disorders [4], most likely due to long-term antimicrobial pressure, frequent veterinary visits [7] and properties of the diseased skin [23], all of which favor the acquisition of MRSP. In light of these facts MRSP can currently be considered more a hospital associated than a community associated pathogen. In animals it may be challenging to differentiate between hospital and community acquired infections. Firstly, there is a lack of common definitions for these in veterinary medicine. Additionally, the majority of hospital acquired infections (such as surgical site infections) appear at home because the duration of hospitalization is usually short and many elective procedures are performed as outpatient surgery (day surgery). Therefore it is uncertain whether infections related to treatment are correctly classified as hospital acquired or veterinary care associated infections. If the spread is not controlled in the veterinary premises, it is inevitable that MRSP will become more prevalent in the community. This increases the likelihood of community acquired MRSP even in animals with no apparent risk factors. Similar development has already been observed in MRSA in humans [2], [24].

There are limitations in this study. The quality of the data is dependent on how well information has been recorded in the patient management software, but this type of bias is expected to be equally distributed among cases and controls. In this study the data were not systematically available on underlying disorders such as allergies or metabolic diseases. In addition, information on variables related to patient care, such as the number of times the animal was handled or the exact placing of the patient (e.g. cage number), was not available. This sort of information could be helpful in order to understand the dynamics of the outbreak. Some degree of misclassification may have occurred due to the imperfect sensitivity of the MRSP screening method. Studies have reported the sensitivity of similar methods for MRSA in humans or livestock to be up to 98% [25]–[28]. Comparable methods have been used for MRSP detection in at least two studies [29], [30], but currently no reference standard for the screening of MRSP exists. Many commercial MRSA selective agars contain cefoxitin as the selective antimicrobial which might impair the growth of MRSP. However, the outbreak strain was highly resistant to oxacillin (MIC >256 µg/ml) and based on our internal evaluation MRSP isolates with oxacillin MIC ≥4 µg/ml are detected even if the bacterial count in the specimen is low. Therefore it is unlikely that a significant amount of misclassification would have occurred. It can be considered a limitation that not all patients could be screened for MRSP upon admission. Consequently, it cannot be ruled out that some of the patients had MRSP already on admission. However, in outbreak situations it is not realistic, nor necessary to screen every patient to determine the admission status. As discussed above, the likelihood of a community acquired MRSP ST71 was very low when all evidence was considered.

In this outbreak no common source for MRSP was identified. Nevertheless, based on the epidemic curve (Figure 2), nosocomial patient-to-patient transmission was likely. It is widely accepted that contaminated hands favor the spread of nosocomial pathogens [31]. In humans the increased use of alcohol based hand rubs has been associated with a decrease in MRSA incidence in hospitals [32], [33]. Barrier nursing has also been shown effective in reducing healthcare-associated MRSA infections [34]. Many hospital pathogens are likely transmitted by fomites, emphasizing the necessity of a clean environment and clothing [35]–[37]. However, the very high number (64 out of 65) of environmental specimens negative for MRSP suggests that the contaminated hospital environment was not the reason for maintenance of the outbreak. The control measures of our outbreak, including cohorting, patient flow planning, emphasis on hand hygiene, barrier nursing, prudent antimicrobial use and environmental cleaning are probably important [31]. Interestingly, Wilson *et al.* [38] found that while the use of enhanced cleaning procedures did reduce the amount of MRSA on hospital surfaces and the hands of healthcare staff, it did not reduce the number of new patients colonized with MRSA, although the authors thought that this may have been a result of a small sample size.

There could be numerous reasons for the long duration of the outbreak. There is evidence that ST71 is capable of efficient dissemination [3], perhaps due to multidrug resistance and a strong ability to form biofilm [19]. While the initial control measures seemed effective at first, it became clear that more rigorous efforts were needed. Initially the lack of resources allocated for infection control was likely to have contributed to the increased number of cases. New employees not familiar with the hygiene practices during the summer of 2011, combined with the lack of personnel due to holidays, may have influenced the increase in incidence. Also, the absence of effective tracking of patients exposed to MRSP until a hygiene nurse was appointed in late 2011 was probably an important factor, since after this the number of new MRSP cases decreased. The cleaning and disinfection at the end of 2011 likely favored the cessation of the outbreak.

In the literature concerning infection control in veterinary hospitals, the importance to recognize patients colonized with multidrug resistant organisms has not been properly acknowledged. Failure to recognize patients with multidrug resistant pathogens – regardless whether infected or colonized – will eventually lead to dissemination of resistant bacteria in hospitals and to the community. The search-and-isolate policy implemented in the Small Animal Hospital is similar to the search-and-destroy policy that has been used to control MRSA in some countries [24], [39]. It does not, however, include the decolonization of patients as no research about the efficacy of decolonization therapy for MRSP has been published. Also, the veterinary use of some antimicrobials used for decolonization of MRSA in humans, such as mupirocin, rifampicin or linezolid, is legally prohibited in Finland. Currently only sporadic cases of MRSP displaying the outbreak antibiogram are identified, mainly among acknowledged risk patients, which indicates the success of present policy.

## Conclusions

We show that multidrug resistant MRSP is capable of causing a large hospital outbreak difficult to control. Our findings suggest that skin lesions of any origin, antimicrobial treatment and prolonged hospital stay increase the probability of acquiring MRSP. We demonstrate that rigorous control measures are needed to control an outbreak and recommend the implementation of a search-and-isolate policy to reduce the burden of MRSP. However, standard precautions (hand disinfection, hygienic work routine, and use of protective clothing in unclean procedures) still remain the core in preventing the transmission of pathogens between patients.

## Supporting Information

Table S1 **Raw data used for the risk factor analysis.**
(XLSX)

File S1 **Assembled sequence of ST71.**
(PDF)

File S2 **Assembled sequence of ST45.**
(PDF)

## References

[1] van Duijkeren E, Catry B, Greko C, Moreno MA, Pomba MC, et al (2011) Review on methicillin-resistant *Staphylococcus pseudintermedius* . J Antimicrob Chemoth 66: 2705–2714. 10.1093/jac/dkr367 21930571

[2] Woodford N, Livermore DM (2009) Infections caused by Gram-positive bacteria: a review of the global challenge. J Infection 59 Suppl 1: S4–16. 10.1016/S0163-4453(09)60003-7 19766888

[3] Perreten V, Kadlec K, Schwarz S, Gronlund Andersson U, Finn M, et al (2010) Clonal spread of methicillin-resistant *Staphylococcus pseudintermedius* in Europe and North America: an international multicentre study. J Antimicrob Chemoth 65: 1145–1154. 10.1093/jac/dkq078 20348087

[4] Huerta B, Maldonado A, Ginel PJ, Tarradas C, Gomez-Gascon L, et al (2011) Risk factors associated with the antimicrobial resistance of staphylococci in canine pyoderma. Vet Microbiol 150: 302–308. 21392899 10.1016/j.vetmic.2011.02.002

[5] Nienhoff U, Kadlec K, Chaberny IF, Verspohl J, Gerlach GF, et al (2011) Methicillin-resistant *Staphylococcus pseudintermedius* among dogs admitted to a small animal hospital. Vet Microbiol 150: 191–197. 21247709 10.1016/j.vetmic.2010.12.018

[6] Weese JS, Faires MC, Frank LA, Reynolds LM, Battisti A (2012) Factors associated with methicillin-resistant versus methicillin-susceptible *Staphylococcus pseudintermedius* infection in dogs. J Am Vet Med Assoc 240: 1450–1455. 22657928 10.2460/javma.240.12.1450

[7] Lehner G, Linek M, Bond R, Lloyd DH, Prenger-Berninghoff E, et al (2014) Case-control risk factor study of methicillin-resistant *Staphylococcus pseudintermedius* (MRSP) infection in dogs and cats in Germany. Vet Microbiol 168: 154–160. 24290489 10.1016/j.vetmic.2013.10.023

[8] Zubeir IE, Kanbar T, Alber J, Lammler C, Akineden O, et al (2007) Phenotypic and genotypic characteristics of methicillin/oxacillin-resistant *Staphylococcus intermedius* isolated from clinical specimens during routine veterinary microbiological examinations. Vet Microbiol 121: 170–176. 17174042 10.1016/j.vetmic.2006.11.014

[9] van Duijkeren E, Houwers DJ, Schoormans A, Broekhuizen-Stins MJ, Ikawaty R, et al (2008) Transmission of methicillin-resistant *Staphylococcus intermedius* between humans and animals. Vet Microbiol 128: 213–215. 18164147 10.1016/j.vetmic.2007.11.016

[10] Clinical and Laboratory Standards Institute (2008) Performance standards for antimicrobial disk and dilution susceptibility tests for bacteria isolated from animals; Approved Standard – 3rd ed. CLSI, Wayne, PA, M31–A3.

[11] Bemis DA, Jones RD, Frank LA, Kania SA (2009) Evaluation of susceptibility test breakpoints used to predict mecA-mediated resistance in *Staphylococcus pseudintermedius* isolated from dogs. J Vet Diagn Invest 21: 53–58. 19139501 10.1177/104063870902100108

[12] Murakami K, Minamide W, Wada K, Nakamura E, Teraoka H, et al (1991) Identification of methicillin-resistant strains of staphylococci by polymerase chain reaction. J Clin Microbiol 29: 2240–2244. 1939577 10.1128/jcm.29.10.2240-2244.1991 PMC270305

[13] Murchan S, Kaufmann ME, Deplano A, de Ryck R, Struelens M, et al (2003) Harmonization of pulsed-field gel electrophoresis protocols for epidemiological typing of strains of methicillin-resistant *Staphylococcus aureus*: a single approach developed by consensus in 10 European laboratories and its application for tracing the spread of related strains. J Clin Microbiol 41: 1574–1585. 12682148 10.1128/JCM.41.4.1574-1585.2003 PMC153895

[14] Tenover FC, Arbeit RD, Goering RV, Mickelsen PA, Murray BE, et al (1995) Interpreting chromosomal DNA restriction patterns produced by pulsed-field gel electrophoresis: criteria for bacterial strain typing. J Clin Microbiol 33: 2233–2239. 7494007 10.1128/jcm.33.9.2233-2239.1995 PMC228385

[15] Kondo Y, Ito T, Ma XX, Watanabe S, Kreiswirth BN, et al (2007) Combination of multiplex PCRs for staphylococcal cassette chromosome mec type assignment: rapid identification system for mec, ccr, and major differences in junkyard regions. Antimicr Agents Ch 51: 264–274. 10.1128/AAC.00165-06 PMC1797693 17043114

[16] Solyman SM, Black CC, Duim B, Perreten V, van Duijkeren E, et al (2013) Multilocus sequence typing for characterization of *Staphylococcus pseudintermedius* . J Clin Microbiol 51: 306–310. 23115265 10.1128/JCM.02421-12 PMC3536184

[17] Sasaki T, Tsubakishita S, Tanaka Y, Sakusabe A, Ohtsuka M, et al (2010) Multiplex-PCR method for species identification of coagulase-positive staphylococci. J Clin Microbiol 48: 765–769. 20053855 10.1128/JCM.01232-09 PMC2832457

[18] Borjesson S, Landen A, Bergstrom M, Andersson UG (2012) Methicillin-Resistant Staphylococcus pseudintermedius in Sweden. Microb Drug Resist 18: 597–603. 22931056 10.1089/mdr.2012.0069

[19] Osland AM, Vestby LK, Fanuelsen H, Slettemeas JS, Sunde M (2012) Clonal diversity and biofilm-forming ability of methicillin-resistant *Staphylococcus pseudintermedius* . J Antimicrob Chemoth 67: 841–848. 10.1093/jac/dkr576 22258925

[20] Lilenbaum W, Nunes EL, Azeredo MA (1998) Prevalence and antimicrobial susceptibility of staphylococci isolated from the skin surface of clinically normal cats. Lett Appl Microbiol 27: 224–228. 9812400 10.1046/j.1472-765x.1998.00406.x

[21] Nienhoff U, Kadlec K, Chaberny IF, Verspohl J, Gerlach G-F, et al (2011) Methicillin-resistant *Staphylococcus pseudintermedius* among cats admitted to a veterinary teaching hospital. Vet Microbiol 153: 414–416. 21703784 10.1016/j.vetmic.2011.05.045

[22] Weese JS (2010) Methicillin-resistant *Staphylococcus aureus* in animals. ILAR J 51: 233–244. 21131724 10.1093/ilar.51.3.233

[23] Simou C, Thoday KL, Forsythe PJ, Hill PB (2005) Adherence of *Staphylococcus intermedius* to corneocytes of healthy and atopic dogs: effect of pyoderma, pruritus score, treatment and gender. Vet Dermatol 16: 385–391. 16359305 10.1111/j.1365-3164.2005.00484.x

[24] Holzknecht BJ, Hardardottir H, Haraldsson G, Westh H, Valsdottir F, et al (2010) Changing epidemiology of methicillin-resistant *Staphylococcus aureus* in Iceland from 2000 to 2008: a challenge to current guidelines. J Clin Microbiol 48: 4221–4227. 20844224 10.1128/JCM.01382-10 PMC3020889

[25] Verkade E, Ferket M, Kluytmans J (2011) Clinical evaluation of Oxoid Brilliance MRSA Agar in comparison with bioMerieux MRSA ID medium for detection of livestock-associated meticillin-resistant *Staphylococcus aureus* . J Med Microbiol 60: 905–908. 21415201 10.1099/jmm.0.021964-0

[26] Verkade E, Verhulst C, van Cleef B, Kluytmans J (2011) Clinical evaluation of Bio-Rad MRSASelect medium for the detection of livestock-associated methicillin-resistant *Staphylococcus aureus* . Eur Journal Clin Microbiol 30: 109–112. 10.1007/s10096-010-1035-7 PMC2998642 20798969

[27] Pletinckx LJ, De Bleecker Y, Dewulf J, Rasschaert G, Goddeeris BM, et al (2012) Evaluation of salt concentrations, chromogenic media and anatomical sampling sites for detection of methicillin-resistant *Staphylococcus aureus* in pigs. Vet Microbiol 154: 363–368. 21890286 10.1016/j.vetmic.2011.07.027

[28] Veenemans J, Verhulst C, Punselie R, van Keulen PH, Kluytmans JA (2013) Evaluation of brilliance MRSA 2 agar for detection of methicillin-resistant *Staphylococcus aureus* in clinical samples. J Clin Microbiol 51: 1026–1027. 23284023 10.1128/JCM.02995-12 PMC3592062

[29] Gomez-Sanz E, Torres C, Lozano C, Saenz Y, Zarazaga M (2011) Detection and characterization of methicillin-resistant *Staphylococcus pseudintermedius* in healthy dogs in La Rioja, Spain. Comp Immunol Microb 34: 447–453. 10.1016/j.cimid.2011.08.002 21903268

[30] Paul NC, Moodley A, Ghibaudo G, Guardabassi L (2011) Carriage of Methicillin-Resistant *Staphylococcus pseudintermedius* in Small Animal Veterinarians: Indirect Evidence of Zoonotic Transmission. Zoonoses Public Health 58: 533–539. 21824350 10.1111/j.1863-2378.2011.01398.x

[31] Weese JS (2012) Staphylococcal control in the veterinary hospital. Vet Dermatol 23: 292–298. 22471691 10.1111/j.1365-3164.2012.01048.x

[32] Lederer JW Jr, Best D, Hendrix V (2009) A comprehensive hand hygiene approach to reducing MRSA health care-associated infections. Jt Comm J Qual Patient Saf 35: 180–185. 19435156 10.1016/s1553-7250(09)35024-2

[33] Sakamoto F, Yamada H, Suzuki C, Sugiura H, Tokuda Y (2010) Increased use of alcohol-based hand sanitizers and successful eradication of methicillin-resistant *Staphylococcus aureus* from a neonatal intensive care unit: a multivariate time series analysis. Am J Infect Control 38: 529–534. 20371134 10.1016/j.ajic.2009.12.014

[34] Perlin JB, Hickok JD, Septimus EJ, Moody JA, Englebright JD, et al (2013) A bundled approach to reduce methicillin-resistant *Staphylococcus aureus* infections in a system of community hospitals. J Healthc Qual 35: 57–68. 23648079 10.1111/jhq.12008

[35] Kramer A, Schwebke I, Kampf G (2006) How long do nosocomial pathogens persist on inanimate surfaces? A systematic review. BMC Infect Dis 6: 130. 16914034 10.1186/1471-2334-6-130 PMC1564025

[36] Boyce JM (2007) Environmental contamination makes an important contribution to hospital infection. J Hosp Infect 65 Suppl 2: 50–54. 17540242 10.1016/S0195-6701(07)60015-2

[37] Singh A, Walker M, Rousseau J, Monteith GJ, Weese JS (2013) Methicillin-resistant staphylococcal contamination of clothing worn by personnel in a veterinary teaching hospital. Vet Surg 42: 643–648. 23662728 10.1111/j.1532-950X.2013.12024.x

[38] Wilson AP, Smyth D, Moore G, Singleton J, Jackson R, et al (2011) The impact of enhanced cleaning within the intensive care unit on contamination of the near-patient environment with hospital pathogens: a randomized crossover study in critical care units in two hospitals. Crit Care Med 39: 651–658. 21242793 10.1097/CCM.0b013e318206bc66

[39] van Trijp MJ, Melles DC, Hendriks WD, Parlevliet GA, Gommans M, et al (2007) Successful control of widespread methicillin-resistant *Staphylococcus aureus* colonization and infection in a large teaching hospital in the Netherlands. Infect Cont Hosp Ep 28: 970–975. 10.1086/519210 17620246

