# Supplementary material for: Large Outbreak Caused by Methicillin Resistant Staphylococcus pseudintermedius ST71 in a Finnish Veterinary Teaching Hospital – From Outbreak Control to Outbreak Prevention
Source: PLoS One. 2014 Oct 15;9(10):e110084. doi: 10.1371/journal.pone.0110084 (PMC4198203; doi:10.1371/journal.pone.0110084)
Supplement: File S1 — Assembled sequence of ST71. (PDF) [file pone.0110084.s002.pdf]

CLC bio

Table of contents

1. Multilocus sequence typing 3
    1.1 Typing results 3
    1.2 Additional information 5

1. Multilocus sequence typing

1.1 Typing results

This report uses the MLST Scheme Staphylococcus pseudintermedius

Allelic Profile and Sequence Type

Gene	Allele sequence	Allelic profile
ack	TCCTCATGTGGCTATATTTGATAC<br>ATCATTCCACCAAACAATGCCTGA<br>ATCAGCATATTTATATAGCTTACCT<br>TATGACTACTATAAAAAATATGGTA<br>TTCGTAAATATGGTTTCCACGGTA<br>CAAGCCATAAATATGTATCACAAC<br>GTGCAGCTGAAATGTTAGGTAAAC<br>CTATTGAGGAATTACGTATTATTTC<br>ATGTCATATTGGTAACGGTGCTTC<br>AATTGCTGCAATCGATGGCGGTAA<br>ATCAATCGACACGTCAATGGGCTT<br>TACACCATTAGCAGGTGTGACAAT<br>GGGTACACGTTCAGGTAATATTGA<br>CCCTGCATTAATTCCATTCTTGAT<br>GGAGAAAACAGGTAAAACGGCTG<br>ATGAAGTACTTAATATTTTAAACAA<br>AGAATCAGGTTTATTAGGTATCAC<br>TGGAACGTCTTCAGATTTACGTGA<br>TATCGAAGACGATGCGAAAAATGG<br>AGAAGAACGTGCAGAGTTAGCGTT<br>AGAAGTATTTGCATCACGCATCCA<br>TAAATACATGGGTTCATATGCAAC<br>ACGTATGCATGGCGTCGATGTGAT<br>CATCTTTAC	3
cpn60	CCCAGTCGGTATCCGCCAAGGTA<br>TTGATAAAGCTGTGGCAGTCGCAA<br>TTGAATCATTACACAATATTTCTCA<br>AAAAGTTGAAAATAAAGAAGAAAT<br>TGCACAAGTCGGCGCGATTTCTG<br>CAGCAGATGAAGAAGTGGGTCGT<br>TACATTTCTGAAGCAATGGAAAAA<br>GTTGGTAATGACGGTGTTATCTCA<br>ATTGAAGAATCGAACGGTTTTAAC<br>ACAGAACTAGAAGTTGTTGAAGGG<br>ATGCAATTTGACCGTGGTTACCAA<br>TCACCATACATGGTGACAGATTCT<br>GACAAAATGATTGCTGAATTAGAA<br>AGACCATACATTTTAATTACTGACA<br>AGAAAATCTCTTCATTCCAAGACA<br>TTTTGCCTTTATTAGAACAAATTGT<br>GCAATCCAACCGTCCAATTTTAAT<br>CGTAGCGGACGAAGTAGAAGGCG	9

Gene	Allele sequence	Allelic profile
fdh	TTCAATTGAACCTGAATCCCCACC<br>ATGACCGACCGTGCGGAATAAAC<br>CTTTTGTCGCTGGTGCTTGGCAAT<br>ATCTTGAACAGTTGTCGATATTGTT<br>CGTACCGATCACTTGGCGTGCAA<br>GTTTTTGCATTAAGTAGGATTCTTC<br>ATTTGTCGCTTTTGAAGATGAAAT<br>GAATGACAATGCATCTGGACCGTA<br>TTGCGCTTTAATTTCTTGCATACGT<br>GTTGCGACATGTTTAATCGCTTCA<br>TCCCATTCGACTTCTTCA	1
pta	ACAGCCGCTGTTGATTTACAAGCT<br>TCTGACTATGTTGCGCCGATCGTA<br>TTAGGGGATGTTGACAAAATTAAA<br>GCACTTGCTGCAGAAAAATCTTTA<br>AATATTGAAGGTTTAAATATCATCC<br>AACCTGATACGAGCGACCTAAAAG<br>CAACACTCGTTGAACAATTTGTAG<br>AACGTCGTAAAGGGAAAGCGACT<br>GAAGAACAAGCACAATCGTTATTA<br>AATGATGTGAACTACTTCGGTACA<br>ATGCTTGTTTATGCAGGTCATGCT<br>GACGGTTTAGTGAGCGGTGCAGC<br>CCACTCAACAGCAGACACTGTTCG<br>TCCAGCGCTTCAAATTATCAAAAC<br>AAAACCAGGTGTTTCTAAGACATC<br>AGGTATTTTCTTCATGATTAAAGAA<br>GATCAACAATTTATTTTCGGTGACT<br>GTGCGATTAACCCTGAATTAGCCG<br>CATCAGACTTAGCTGAAATTGCGG<br>TTGAAAGCGCGAAAACTGCACAAA<br>GCTTCGGCATG	2
purA	TATGCAGCTGGTCAACGTCTTGCA<br>CCATACGTCACAGATACTGCAAAA<br>GTGTTAGACGATGCTTTCGTAGCA<br>GATGAACGTGTATTATTTGAAGGT<br>GCACAAGGTGTGATGTTAGATATC<br>GACCACGGCACATATCCTTTCGTT<br>ACATCAAGCAATCCTGTCGCAGGT<br>AACGTCACTGTAGGCGCGGGTGT<br>CGGCCCAACAAACGTTTCAAAAGT<br>TGTCGGTGTATGTAAAGCGTATAC<br>GTCACGTGTAGGTGATGGCCCGT<br>TCCCAACAGAATTATTTGACGAAA<br>AAGGTCATCATATCCGTGAAGTAG<br>GTCGCGAATATGGTACAACAACAG<br>GTCGTCCGCGTCGTGTCGGCTGG<br>TTTGACTCAGTGGTGTTACGTCAC<br>TCTCGTCGTGTGAGTGGGATTACT	1

Gene	Allele sequence	Allelic profile
sar	GTTAAAAGGTGACGACTTATTCGG TACAGGAAATGCATTGAAGTTAGC CTTTAGTGGTTTTTCAACGAGTGC CGTCGCGTTAGTTGCTGCAGCATT GTTCTTAGCAACCGCGATGCAAGT GACAAACTTACATAAACGTTTGGC ACTATTAGTATTATCGTTTGTCGGT AATAAAACGAAAAATATCGTTATC GGGGCGATTCTTGTTTCTATTATTT TAGCGTTTTTCGTACCGTCCGCTA CAGCACGTGCTGGCGCTGTTGTA CCGATTTTATTAGGTATGATTGCG GCGTTTGGGGCAACGAAAAATAGT AAATTAGCTGCGTTACTCATCATTA CAGCTGTACAAGCGGTATCGATTT GGAATATCGGGATT	2
tuf	TGAGCACATTCTTTTATCACGTAA CGTTGGTGTACCAGCATTAGTTGT ATTCTTAAACAAAGTTGACATGGT AGACGACGAAGAATTATTAGAATT AGTAGAAATGGAAGTACGTGACTT ATTATCTGAATACGACTTCCCAGG TGATGACGTACCTGTAATCGCTGG TTCAGCATTAAAAGCTTTAGAAGG CGATGCACAATACGAAGAAAAAAT CTTAGAATTAATGGAAGCTGTAGA CACTTACATTCCAACTCCAGACCG TGACTCAGACAAACCATTCATGAT GCCTGTTGAGGACGTATTCTCAAT CACAGGTCGTGGTACAGTTGCTAC TGGTCGTGTTGAACGTGGTCAAAT CAAAGTTGGTGACGAAGTAGAAAT CATCGGTTTAACTGAAGAATCTTC TAAAACAAC	1

ack	cpn60	fdh	pta	purA
3	9	1	2	1

sar	tuf
2	1

Sequence Type: 71;

1.2 Additional information

For more information about MLST and other MLST Schemes visit the homepages:
PubMLST <http://pubmlst.org/>
MLST.net <http://www.mlst.net/>
MLST Databases at the ERI, University College Cork <http://mlst.ucc.ie/>
