# Supplementary material for: Large Outbreak Caused by Methicillin Resistant Staphylococcus pseudintermedius ST71 in a Finnish Veterinary Teaching Hospital – From Outbreak Control to Outbreak Prevention
Source: PLoS One. 2014 Oct 15;9(10):e110084. doi: 10.1371/journal.pone.0110084 (PMC4198203; doi:10.1371/journal.pone.0110084)
Supplement: File S2 — Assembled sequence of ST45. (PDF) [file pone.0110084.s003.pdf]

CLC bio

Table of contents

1. Multilocus sequence typing 3
1.1 Typing results 3
1.2 Additional information 5

1. Multilocus sequence typing

1.1 Typing results

This report uses the MLST Scheme Staphylococcus pseudintermedius

Allelic Profile and Sequence Type

Gene	Allele sequence	Allelic profile
ack	TCCTCATGTGGCTATATTTGATAC<br>ATCATTCCACCAAACAATGCCTGA<br>ATCAGCATATTTATATAGCTTACCT<br>TATGACTACTATAAAAAATATGGTA<br>TTCGTAAATATGGTTTCCACGGTA<br>CAAGCCATAAATATGTATCACAAC<br>GTGCAGCTGAAATGTTAGGTAAAC<br>CTATTGAGGAATTACGTATTATTTC<br>ATGTCATATTGGTAACGGTGCTTC<br>AATTGCTGCAATCGATGGCGGTAA<br>ATCAATCGACACGTCAATGGGCTT<br>TACACCATTAGCAGGTGTGACAAT<br>GGGTACACGTTCAGGTAATATTGA<br>CCCTGCATTAATTCCATTCTTGAT<br>GGAGAAAACAGGTAAAACGGCTG<br>ATGAAGTACTTAATATTTTAAACAA<br>AGAATCAGGTTTATTAGGTATCAC<br>TGGAACGTCTTCAGATTTACGTGA<br>TATCGAAGACGATGCGAAAAATGG<br>AGAAGAACGTGCAGAGTTAGCGTT<br>AGAAGTATTTGCATCACGCATCCA<br>TAAATACATGGGTTCATATGCAAC<br>ACGTATGCATGGCGTCGATGTGAT<br>CATCTTTAC	3
cpn60	CCCAGTCGGTATCCGCCAAGGTA<br>TTGATAAAGCTGTGGCAGTCGCAA<br>TTGAATCATTACACAATATTTCTCA<br>AAAAGTTGAAAATAAAGAAGAAAT<br>TGCACAAGTCGGCGCGATTTCTG<br>CAGCAGATGAAGAAGTGGGTCGT<br>TACATTTCTGAAGCAATGGAAAAA<br>GTTGGTAATGACGGTGTTATCTCA<br>ATTGAAGAATCGAACGGTTTCAAC<br>ACAGAACTAGAAGTTGTTGAAGGG<br>ATGCAATTTGACCGTGGCTACCAA<br>TCGCCATACATGGTGACAGATTCT<br>GACAAAATGATTGCTGAATTAGAA<br>AGACCATACATTTTAATTACTGACA<br>AGAAAATCTCTTCATTCCAAGACA<br>TCTTGCCTTTATTAGAGCAAATTGT<br>ACAATCAAACCGTCCAATTTTAATC<br>GTAGCGGACGAAGTAGAAGGCG	2

Gene	Allele sequence	Allelic profile
fdh	TTCAATTGAACCTGAATCCCCACC<br>ATGACCGACCGTGCGGAATAAAC<br>CTTTTGTCGCTGGTGCTTGGCAAT<br>ATCTTGAACAGTTGTCGATATTGTT<br>CGTACCAATTACTTGACGTGCAAG<br>TTTTTGCATTAAGTAGGATTCTTCA<br>TTTGTCGCTTTTGAAGATGAAATG<br>AATGACAATGCATCTGGACCGTAT<br>TGCGCTTTAATTTCTTGCATACGT<br>GTTGCGACATGTTTAATCGCTTCA<br>TCCCATTCGACTTCTTCA	2
pta	ACAGCCGCTGTTGATTTACAAGCT<br>TCTGACTATGTTGCGCCGATCGTA<br>TTAGGGAATGTTGACAAAATTAAA<br>GCACTTGCTGCAGAAAAATCTTTA<br>AATATTGAAGGTTTAAATATCATCC<br>AACCTGATACGAGCGACCTAAAAG<br>CAACACTCGTTGAACAATTTGTAG<br>AACGTCGTAAAGGGAAAGCGACT<br>GAAGAACAAGCACAATCGTTATTA<br>AATGATGTGAACTACTTCGGTACA<br>ATGCTTGTTTATGCAGGTCATGCT<br>GACGGTTTAGTGAGCGGTGCAGC<br>CCACTCAACAGCAGACACTGTTCG<br>TCCAGCGCTTCAAATTATCAAAAC<br>AAAACCAGGTGTTTCTAAGACATC<br>AGGTATTTTCTTCATGATTAAAGAA<br>GATCAACAATTTATTTTCGGTGACT<br>GTGCGATTAACCCTGAATTAGCCG<br>CATCAGACTTAGCTGAAATTGCGG<br>TTGAAAGCGCGAAAACTGCACAAA<br>GCTTCGGCATG	1
purA	TATGCAGCTGGTCAACGTCTTGCA<br>CCATACGTCACAGATACTGCAAAA<br>GTGTTAGACGATGCTTTCGTAGCA<br>GATGAACGTGTATTATTTGAAGGT<br>GCACAAGGTGTGATGTTAGATATC<br>GACCACGGCACATATCCTTTCGTT<br>ACATCAAGCAATCCTGTCGCAGGT<br>AACGTCACTGTAGGCGCTGGTGTT<br>GGCCCAACAAACGTTTCAAAAGTT<br>GTCGGTGTATGTAAAGCGTATACG<br>TCACGTGTAGGTGATGGCCCGTT<br>CCCAACAGAATTATTTGACGAAAA<br>AGGTCATCATATCCGTGAAGTAGG<br>TCGCGAATATGGTACAACAACAGG<br>TCGTCCGCGTCGTGTCGGCTGGT<br>TTGACTCAGTGGTGTTACGTCACT<br>CTCGTCGTGTGAGTGGGATTACT	2

Gene	Allele sequence	Allelic profile
sar	GTTAAAAGGTGACGACTTATTCGG<br>TACAGGAAATGCATTGAAGTTAGC<br>CTTTAGTGGTTTTTCAACGAGTGC<br>CGTCGCGTTAGTTGCTGCAGCATT<br>GTTCTTAGCAACCGCGATGCAAGT<br>GACAAACTTACATAAACGTTTGGC<br>ACTATTAGTATTATCGTTTGTCGGT<br>AATAAAACGAAAAATATCGTTATC<br>GGGGCGATTCTTGTATCTATTATT<br>TTAGCGTTTTTCGTACCGTCCGCT<br>ACAGCACGTGCTGGCGCTGTTGT<br>ACCGATTTTATTAGGTATGATTGC<br>GGCGTTTGGGGCAACGAAAAATA<br>GTAAATTAGCTGCGTTACTCATCA<br>TTACAGCTGTACAAGCGGTATCGA<br>TTTGGAATATCGGGATT	1
tuf	TGAGCACATTCTTTTATCACGTAA<br>CGTTGGTGTACCAGCATTAGTTGT<br>ATTCTTAAACAAAGTTGACATGGT<br>AGACGACGAAGAATTATTAGAATT<br>AGTAGAAATGGAAGTACGTGACTT<br>ATTATCTGAATACGACTTCCCAGG<br>TGATGACGTACCTGTAATCGCTGG<br>TTCAGCATTAAAAGCTTTAGAAGG<br>CGATGCACAATACGAAGAAAAAAT<br>CTTAGAATTAATGGAAGCTGTAGA<br>CACTTACATTCCAACTCCAGACCG<br>TGACTCAGACAAACCATTCATGAT<br>GCCTGTTGAGGACGTATTCTCAAT<br>CACTGGTCGTGGTACAGTTGCTAC<br>TGGTCGTGTTGAACGTGGTCAAAT<br>CAAAGTTGGTGACGAAGTAGAAAT<br>CATCGGTTTAACTGAAGAATCTTC<br>TAAAACAAC	2

ack	cpn60	fdh	pta	purA
3	2	2	1	2

sar	tuf
1	2

Sequence Type: 45;

1.2 Additional information

For more information about MLST and other MLST Schemes visit the homepages:
PubMLST <http://pubmlst.org/>
MLST.net <http://www.mlst.net/>
MLST Databases at the ERI, University College Cork <http://mlst.ucc.ie/>
